# Efficacy of oxidized regenerated cellulose combined with fibrin glue in reducing pulmonary air leakage after segmentectomy in a porcine lung model

**DOI:** 10.3389/fbioe.2022.1052535

**Published:** 2022-12-05

**Authors:** He Yang, Zhiqiang Dong, Hongya Wang, Zicheng Liu, Wenbo Sun, Kun Wang, Xinfeng Xu, Wei Wen, Jun Wang, Liang Chen, Quan Zhu

**Affiliations:** ^1^ Department of Thoracic Surgery, The First Affiliated Hospital of Nanjing Medical University, Nanjing, China; ^2^ Department of Cardiovascular Surgery, The First Affiliated Hospital of Nanjing Medical University, Nanjing, China; ^3^ Department of Thoracic Surgery, Nanjing Chest Hospital, Nanjing, China

**Keywords:** segmentectomy, pulmonary air leakage, oxidized regenerated cellulose, fibrin glue, polyglycilic acid sheet

## Abstract

**Objectives:** Pulmonary air leakage is a common complication following lung resection. We have designed a new method combining oxidized regenerated cellulose and fibrin glue to cover the intersegmental plane in clinical lung segmentectomy to prevent postoperative air leakage. In this study, an excised porcine lung segmentectomy model was created to validate its adhesive strength and effect on reducing air leakage.

**Methods:** In the pre-experiment, six different larger lung segments were separated using electrocautery on the fresh isolated porcine lungs (*n* = 5 in each group). The air leakage degree and operation time of the lung segments were comprehensively evaluated to select the most suitable target segment for establishing the *ex vivo* porcine lung segmentectomy models. In the experiment, according to the different materials covered on the intersegmental plane, these models were randomly divided into four groups: group A used fibrin glue and oxidized regenerated cellulose (ORC) mesh (*n* = 20); group B used fibrin glue and polyglycolic acid (PGA) sheet (n = 20); group C used fibrin glue (*n* = 20); group D was the blank control group (*n* = 20). The minimum air leakage pressure (MALP) of the selected target segment in each group was measured using a stepwise increase of airway pressure, and histological assessment was performed on the sealed area samples from the four groups.

**Results:** The operation time of the a segment of the right cranial lobe (R1a) was shorter than that of other segments (*p* < 0.05), and there was no significant difference in the air leakage pressures between the six isolated segments (*p* = 0.76); thus, R1a was chosen for segmentectomy. In addition, the MALP was significantly higher in group A (41.8 ± 4.5 cmH_2_O) than in groups C (28.1 ± 2.3 cmH_2_O) and D (17.3 ± 1.2 cmH_2_O) (both *p* < 0.001). The MALP of group B (69.5 ± 5.2 cmH_2_O) was significantly higher than that of group A (*p* < 0.001), whereas that of group C was significantly higher than that of group D (*p* < 0.001). Histological examination confirmed that the combined use of fibrin glue and ORC or PGA patch adhered more firmly to the intersegmental plane than that of fibrin glue alone, although some gaps could be seen between the fibrin glue and the surface of the lung segments in group C.

**Conclusion:** The application of ORC combined with fibrin glue on the intersegmental plane has a good sealing performance in the *ex vivo* porcine lung segmentectomy model, suggesting that ORC may be an effective alternative material to replace PGA sheet to combine with fibrin glue for preventing air leakage after segmentectomy.

## Introduction

Recently, precise anatomical segmentectomy is increasingly being used as an important option for treating early non-small cell lung cancer to preserve more effective lung tissue without compromising oncologic outcomes (Leshnower et al., 2010). However, the incidence of pulmonary air leakage after segmentectomy is higher than that for lobectomy (Suzuki et al., 2019) because air leakage occurs easily when using electrocautery, ultrasonic scalpel, or stapler to cut the intersegmental plane (Yazawa et al., 2021; Saito et al., 2017).

Studies have shown that polyglycolic acid (PGA) mesh and fibrin glue can be used to cover the pleura defect, which can effectively reduce postoperative pulmonary air leakage (Gika et al., 2007; Ueda et al., 2007; Kawai et al., 2012; Matoba et al., 2018; Tanaka et al., 2018). However, most animal experiments have only focused on the peripheral pleural defects caused by artificial damage, such as needle puncture (Matoba et al., 2018; Gika et al., 2007). Furthermore, this method may be applicable to wedge resection and lobectomy even though the intersegmental plane has not been well studied, thus suggesting an inconsistency with clinical segmentectomy. However, the high cost of PGA patches in lung surgery may increase hospital costs (Budak et al., 2020). In addition, several studies have shown that PGA degradation can cause inflammation (Ceonzo et al., 2006; Yang et al., 2005), and severe thoracic adhesion can be caused by the PGA felt (Nakamura et al., 2010; Saito et al., 2017). Therefore, we tried to find a substitute for the PGA mesh and design a new experimental method to investigate the adhesion properties of various materials in segmentectomy.

Oxidized regenerated cellulose (ORC) is a bio-absorbable material obtained by controlled chemical oxidation of cellulose. It is often used in surgical operation because of its low price, strong hemostatic properties, and good biocompatibility (Lewis et al., 2013; Martyn et al., 2015; Khoshmohabat et al., 2019). Moreover, the application of the ORC mesh to the staple line effectively reduces the postoperative recurrence rate after bullectomy (Lee et al., 2006; Cho et al., 2008; Lee et al., 2014). We pioneered the combined use of ORC and fibrin glue in segmentectomy performed at our center, and we observed that the incidence of postoperative pulmonary air leakage was low; however, whether ORC could enhance the adhesive strength of fibrin glue has not been confirmed.

In this study, a porcine lung model was created to test the adhesive quality of the combined application of fibrin glue and ORC in the intersegmental plane. Histological examination was also performed to confirm the adhesive strength and explore whether ORC can replace PGA mesh in segmentectomy.

## Materials and methods

### Porcine lungs

Isolated bilateral porcine lungs (purchased from Jiangsu Jurong Kangrong Poultry Industry Co., Ltd.) were obtained from healthy domestic 6-month-old pigs weighing (100 ± 25) kg, which were slaughtered on the same day. The lungs were excluded from the experiment when there were bullae, severe congestion, infection, damage, anatomical variation, or other factors that might influence the experimental results. To maintain the physiological state of the lungs and avoid the loss of pulmonary surfactant as much as possible, all experiments were completed within 4 h after the lung was isolated.

### Tissue sealants

Fibrin glue (Beixiu®, Porcine Fibrin Sealant Kit, Guangzhou, China) is a synthetic absorbable biomaterial comprising main glue and catalyst. The main glue contains fibrinogen and coagulation factor XIII. The catalyst is a solution containing thrombin and CaCl_2_. When the two solutions are mixed, simulating the last stage of the blood coagulation process, a stable fibrin polymer is formed, which presents a cross-multi-layered and homogeneous mesh structure that can trap erythrocytes and the active ingredients in plasma and provide biological scaffolds for the growth of fibroblasts and capillaries to seal and repair the pleura. Moreover, as a flexible mechanical leakage barrier, it is durable for 14 days, making the tissue of the treatment site expand completely and promoting wound healing. Once the fibrin glue is applied, it will be degraded by hydrolysis, releasing biocompatible ingredients which are metabolized or removed by the kidneys.

ORC (Ethicon Surgicel® absorbable hemostat mesh, Johnson & Johnson, NJ, United States), which can be trimmed to any shape, makes full contact with human tissue easily because of its fabric-like property. Furthermore, being a cellulose derivative, it has good biocompatibility, biodegradability, and low toxicity. Its hemostatic function involves coagulating the wound’s blood on mesh gauze through physical action, promoting platelet rupture, producing many platelet coagulation factors, and converting fibrinogen to fibrin to form a thrombus. At the same time, the hydroxyl group in cellulose forms acid-hematin with Fe^2+^ in plasma, a black gel-like substance that aids hemostasis (Wu et al., 2012). Moreover, Surgicel® is almost completely absorbed by the human body in 7 days, without causing adverse reactions to the surrounding tissues.

Absorbable PGA patch (Neville®, Gunze Ltd., Kyoto, Japan) is made of a loose and highly elastic PGA sheet with a fiber diameter of 16.1 nm, an average distance of 27.4 nm between fibers, and an average thickness of 0.15 mm. Neville® has two variants—the tubular type and the flake type. The flake type was used in this study. The human body completely absorbs the sheet in approximately 15 weeks‬‬‬‬‬‬‬‬‬‬.

### Surgical procedure

**FIGURE 1:**
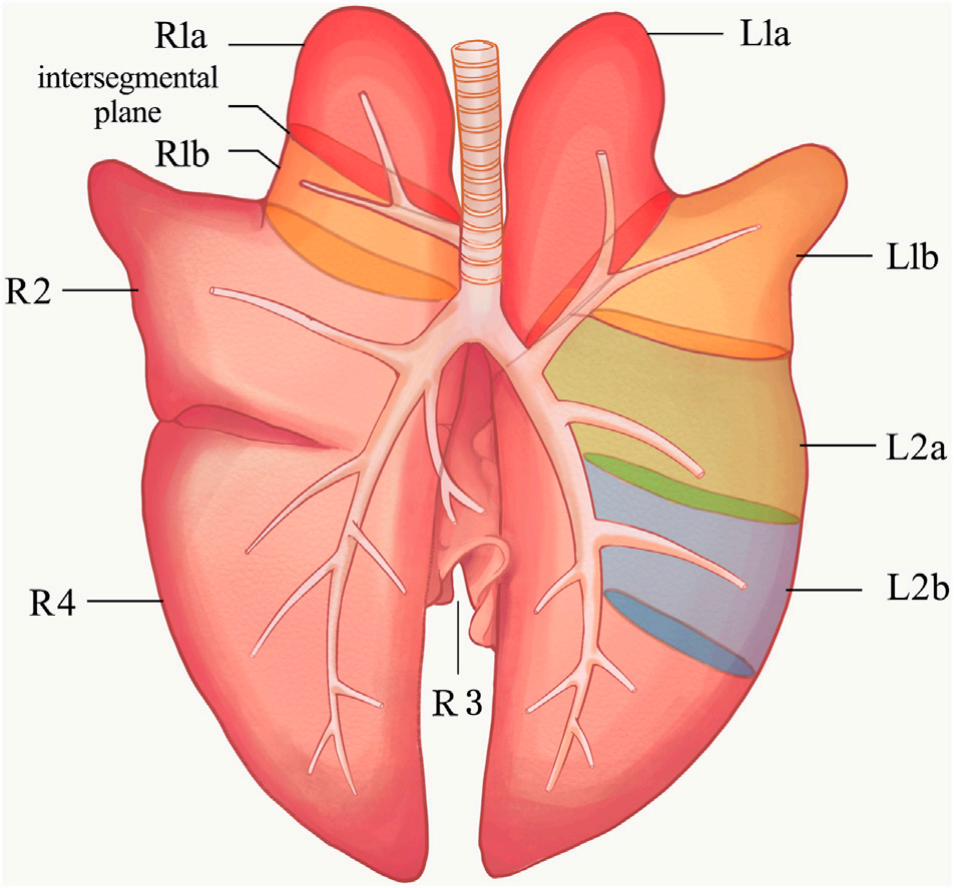
Anatomical diagram of porcine lung. L1, left cranial lobe; L2, left caudal lobe; R1, right cranial lobe; R2, right middle lobe; R3, right accessory lobe; R4, right caudal lobe. The ventilation of each lung segment is controlled by its corresponding bronchus. The bronchus of R1 opens directly to the main trachea. The R1a, R1b, L1a, L1b, L2a, and L2b segments were used in the pre-experiments, and finally R1a was selected for the segmentectomy, the intersegmental plane was the location of the sealant application in the experiments. R1a, R1b (a and b segments of the right cranial lobe); L1a, L1b (a and b segments of the left cranial lobe); L2a, L2b (a and b segments of the left caudal lobe).

To determine the suitable lung segment for this experiment, we performed a pre-experiment on six large porcine lung segments to compare the surgical separation time, and the respective air leakage pressures were evaluated. As shown in Figure 1, the ventilation of each lung segment is controlled by its corresponding bronchus. Therefore, the a and b segments of the right cranial lobe (R1a, R1b), the a and b segments of the left cranial lobe (L1a, L1b), and the a and b segments of the left caudal lobe (L2a, L2b) were used in the pre-experiments.

**FIGURE 2:**
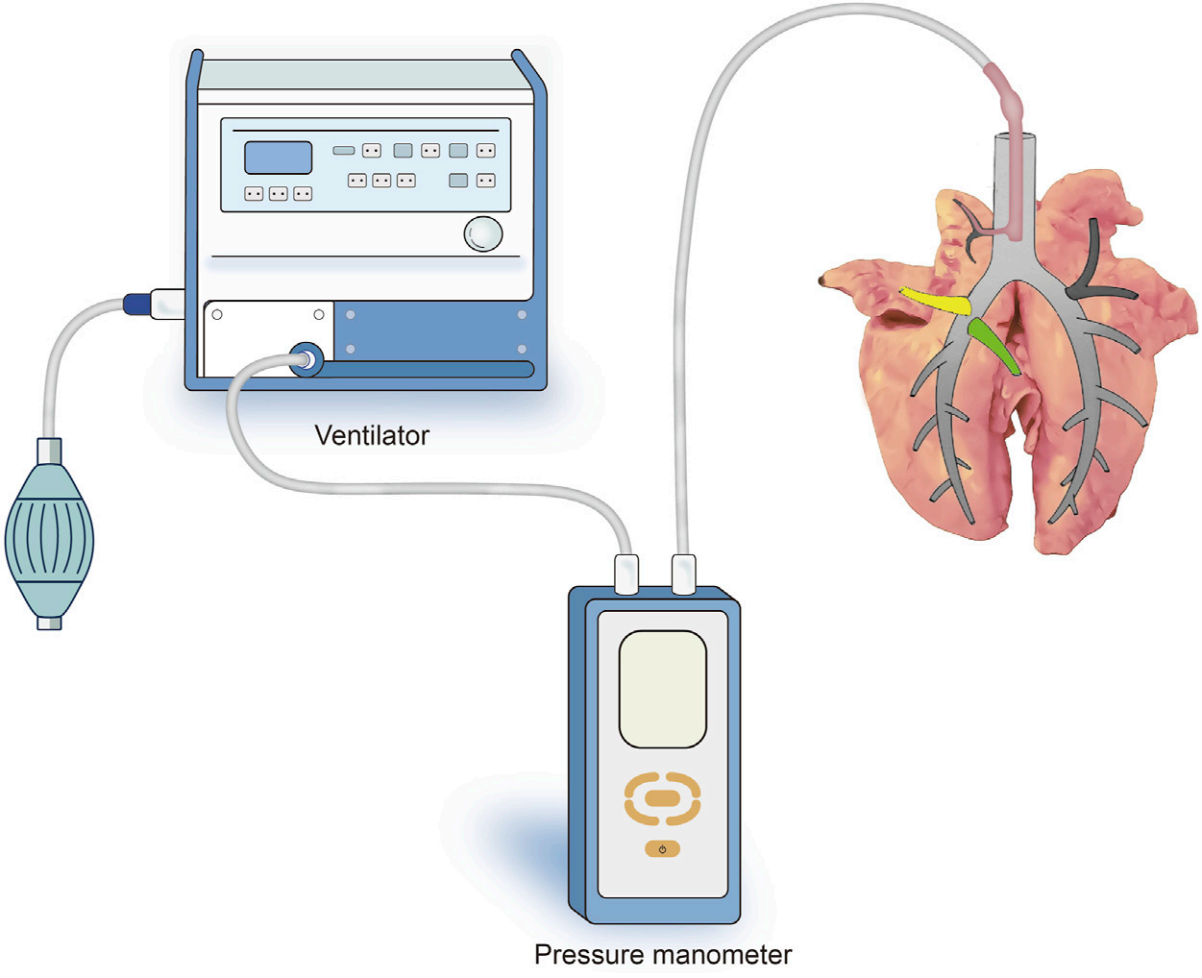
Schematic representation of the experimental setup for ventilating the target segment and measuring the pressure in the airway. The 12-Fr cuffed endotracheal tube attached to the mechanical ventilator was inserted into the six segmental bronchi sequentially to ventilate the target segment and the cuff was inflated to ensure air tightness (At this point, the endotracheal tube was inserted into the R1a bronchus).

**FIGURE 3:**
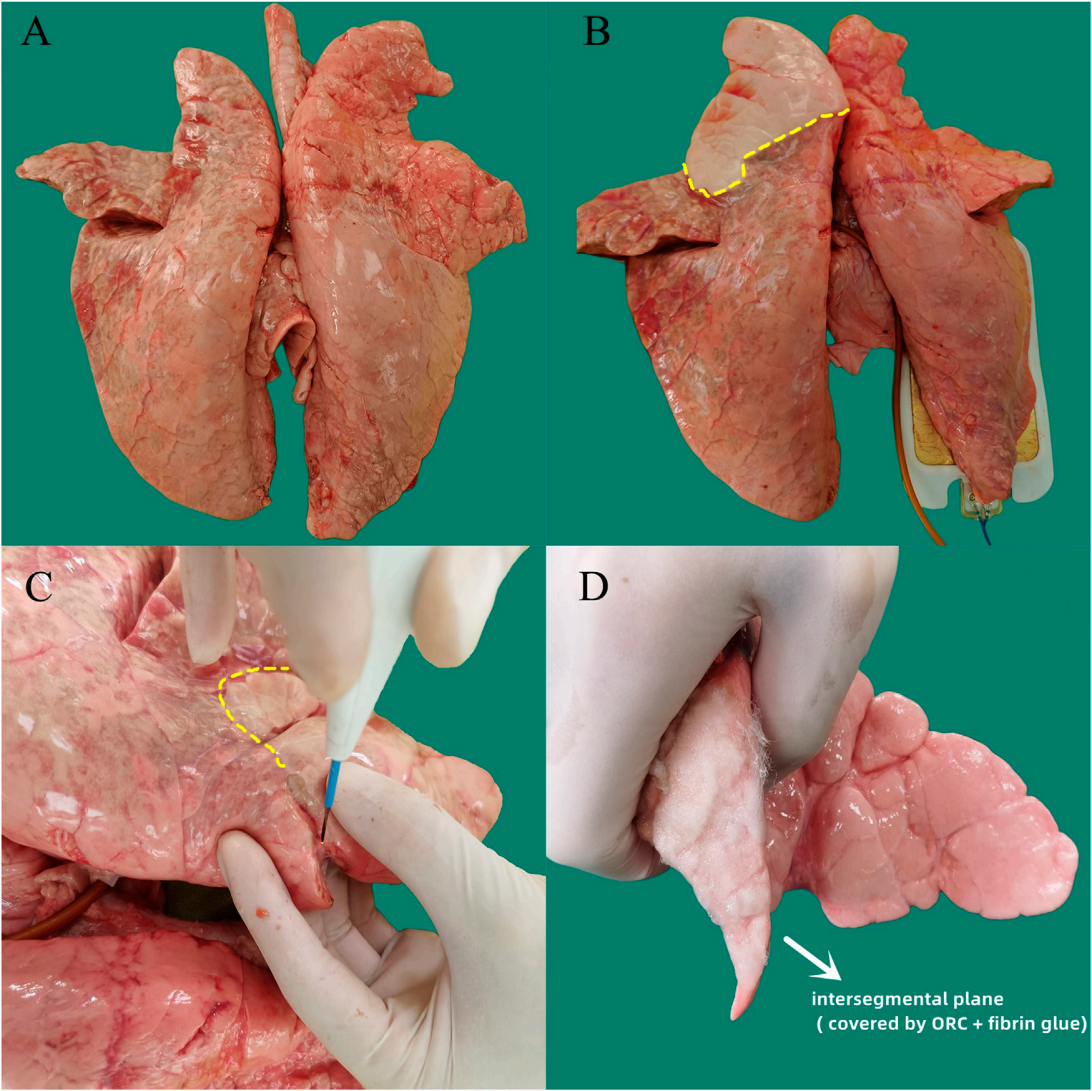
Experimental procedure for separating the target segment and covering the intersegmental plane with sealing materials in the *ex vivo* porcine lung segmentectomy model. **(A)** Normal isolated bilateral porcine lungs. **(B)** The target segment was obviously inflated by the ventilation, with a stable intersegmental interface (yellow dotted line) between it and the surrounding uninflated lung tissue of the adjacent lung segfment. **(C)** An electric cautery was used as the energy device to separate the intersegmental plane (yellow dotted line), and scalpel was used to cut off the hilar connective tissue and blood vessels. **(D)** ORC sheet and fibrin glue were conbined using the method of group A (ORC + fibrin glue) to seal the intersegmental plane. ORC (oxidized regenerated cellulose).

After sputum aspiration in the bronchus, a 12-Fr cuffed endotracheal tube attached to the mechanical ventilator (Aestiva/5 7,100, Datex-Ohmeda, Madison, WI, United States ) was inserted into the six segmental bronchi sequentially and the cuff was inflated to ensure air tightness. Subsequently, the lungs were sunk into a tank of saline solution kept at a temperature of 37°C, and the target segment was slowly inflated in water by the ventilator. A micromanometer (BENETECH, Jumaoyuan Technology Co., Ltd., Shenzhen, China, GM520, ±35 kPa, ±0.3%) was used to measure the air leak pressure (Figure 2). To avoid alveolar structural damage caused by overinflation, the inflation pressure was controlled to less than 20 cmH_2_O. The lung was abandoned if the target lung was difficult to expand or leak. Using this stepwise increase of airway pressure method, we observed that the target lung was obviously expanded, with a stable intersegmental interface between it and the uninflated lung tissue of the adjacent segment, and the boundary line was marked (Figures 3A,B).

When separating the intersegmental interface, an electric cautery was used as the energy device in the coagulation mode at a power of 40 W (POWER-420B, Changzhou Yanling Electronic equipment Co., Ltd., Changzhou, China) (Figure 3C). During the separation process, the intersegmental interface was carefully identified to avoid misoperation, and the lung was discarded in case of wrong segmentation. Then, sharp instruments such as scalpel were used to cut off the hilar connective tissue and blood vessels, and the target bronchus was finally cut off to complete the segmentectomy. The operation time for the surgical separation of the six lung segments was recorded.

### Measurement of the pulmonary air leak pressure

The micromanometer and ventilator were connected to the target lung segments by the soft catheter. Then the segments were immersed in a physiologic saline tank maintained at 37°C and airway pressure of 10 cmH_2_O. The ventilation started with 10 cmH_2_O, and stepwise increments of 5 cmH_2_O per minute were applied up to 70 cmH_2_O, which is the maximum pressure of the ventilator. The ventilator settings were as follows: pressure control ventilation, (respiratory frequency 12; inspiration-to-expiration ratio = 1:2; positive end-expiratory pressure 0). The intratracheal pressure was measured using a manometer attached to the ventilator through a tracheal tube. The minimum air leakage pressure (MALP) was defined as the pressure at the emersion of the bubble flow from the intersegmental plane under water. If the sealants persisted to the maximum pressure setting of the ventilator, an additional pressure of 70 cmH_2_O was applied manually using a ventilation bag at the speed of 2 cmH_2_O/min until air leakage occurred. Two experimenters independently observed and recorded the MALP. The values were retested and read if the date deviation was more than 1 cmH_2_O. However, the lung segment was abandoned if the deviation was more than 1 cmH_2_O again. The average values of the dates recorded by the two experimenters were taken as the final results.

The operation time and MALP of the six lung segments were comprehensively evaluated to select the most suitable target segment for establishing the *ex vivo* porcine lung segmentectomy model.

### Randomized grouping

**FIGURE 4:**
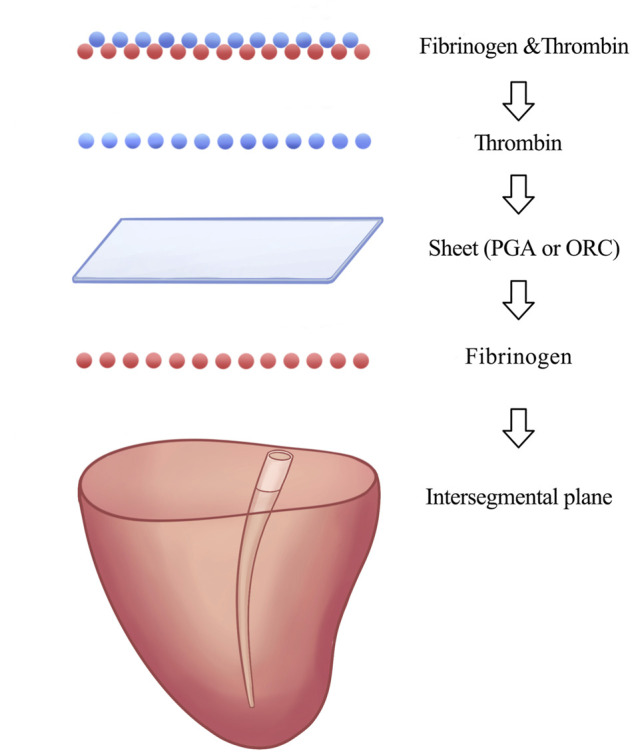
Sealing material application procedures of pack method in experimental groups **(A)** and **(B)**.

To avoid selection bias, porcine segmentectomy models were randomly divided into four groups. Group A: 0.25 ml of the liquid main glue was sprayed at the intersegmental plane, then the ORC sheet (5.1 × 10.2 cm^2^, thickness of 2 mm) was placed over the interface after cutting off the extra parts of the periphery, and 0.25 ml of the liquid catalyst solution was sprayed on the sheet, followed by 0.25 ml of the liquid main glue and 0.25 ml of the liquid catalyst (*n* = 20) (Figure 3D). Group B: 0.25 ml of the liquid main glue was sprayed at the intersegmental plane, and then the PGA sheet (100 × 50, 0.15 mm in thickness) was covered on the interface. The peripheral excess was cut off, and 0.25 ml of the liquid catalyst solution was sprayed on the sheet, followed by 0.25 ml of liquid main glue and 0.25 ml of the liquid catalyst (n = 20). Group C: 0.5 ml of the liquid main glue was sprayed on the intersegmental interface, followed by 0.5 ml of the liquid catalyst (n = 20). Group D did not receive any intervention at the intersegmental plane (n = 20). The pack method we used to apply the sealing materials in groups A and B was shown in Figure 4. The lung segmentectomy models in each group were allowed to stand for 5 min to fix the sealing materials, and their MALPs were measured using the method mentioned above, similar to the measurement of the pulmonary air leak pressure.

### Histological assessment

The pulmonary segmentectomy models of groups A, B, C, and D were obtained in the same preparation manner as that for the pressure resistance test, then the lung tissue in the center of the intersegmental plane covered with the sealing materials was resected for histologic assessment (1 × 1 cm). The specimens were fixed in 10% formalin, embedded in paraffin, and sectioned for hematoxylin-eosin staining. We examined at least five different sites in each specimen.

### Statistical analysis

SPSS version 24.0 (SPSS Inc., Chicago, IL, United States ) software package was used for all statistical analyses. All data are expressed as the mean ± standard deviation. One-way analysis of variance and Tukey’s test were used to compare the data among the groups. Statistical significance was set at *p* < 0.05.

## Results

### Operation time and pressure resistance in the pre-experiment

**FIGURE 5:**
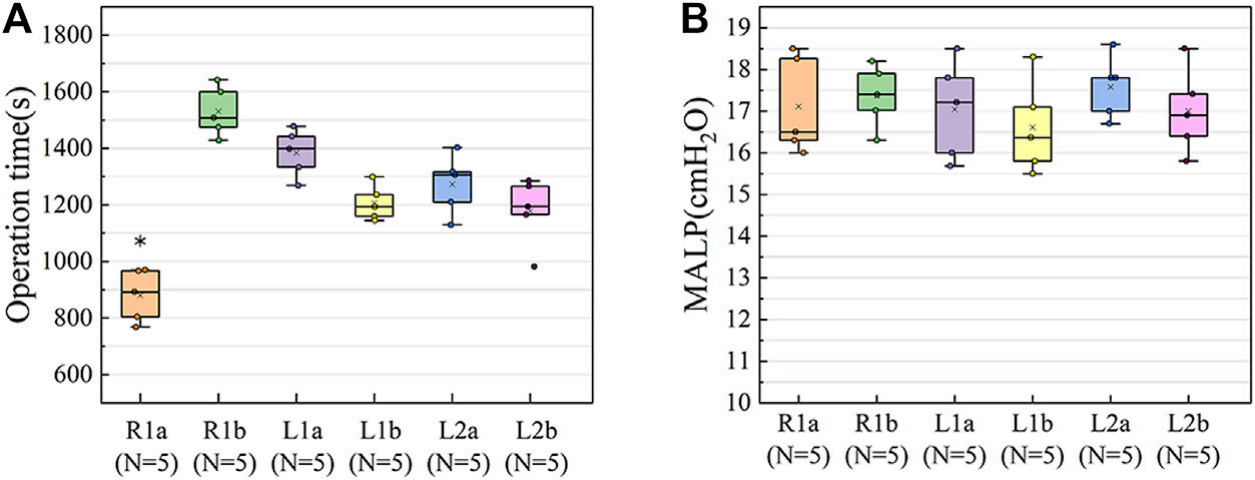
Results of the pre-experiments. **(A)** The operation time of R1a was shorter than in the other groups (F = 27.26, *p* < 0.05). **(B)** The difference in the MALP between the six groups of models was not statistically significant (F = 0.519, *p* = 0.76). The upper and lower borders of the box represent, the upper and lower quartiles. The middle horizontal line represents the median. The cross represents the mean. The whiskers show the minimum and maximum values excluding the outliers. *p < 0.05, the operation time of R1a *versus* the other lung segments. R1a, R1b (a and b segments of the right cranial lobe); L1a, L1b (a and b segments of the left cranial lobe); L2a, L2b (a and b segments of the left caudal lobe); MALP (minimum air leakage pressure).

The average operation times of the different segmentectomy models were as follows: R1a (880 ± 83) s; R1b (1531 ± 79) s; L1a (1384 ± 75) s; L1b (1207 ± 56) s; L2a (1273 ± 94) s; L2b (1179 ± 108) s. The operation time of R1a was shorter than that of other groups (F = 27.26, *p* < 0.05) (Figure 5A), this may be because the bronchus of R1 opens directly to the main trachea. However, the difference in the MALP between the six models was not statistically significant (F = 0.52, *p* = 0.76) (Figure 5B). The MALPs measured in the lung segment models were as follows: R1a (17.1 ± 1.1) cmH_2_O; R1b (17.4 ± 0.7) cmH_2_O; L1a (17.0 ± 1.1) cmH_2_O; L1b (16.6 ± 1.0) cmH_2_O, L2a (17.6 ± 0.7) cmH_2_O, and L2b (17.0 ± 0.9) cmH_2_O. Due to the short operation time, R1a was chosen for segmentectomy.

### Pressure resistance of the porcine lung segmentectomy models

**FIGURE 6:**
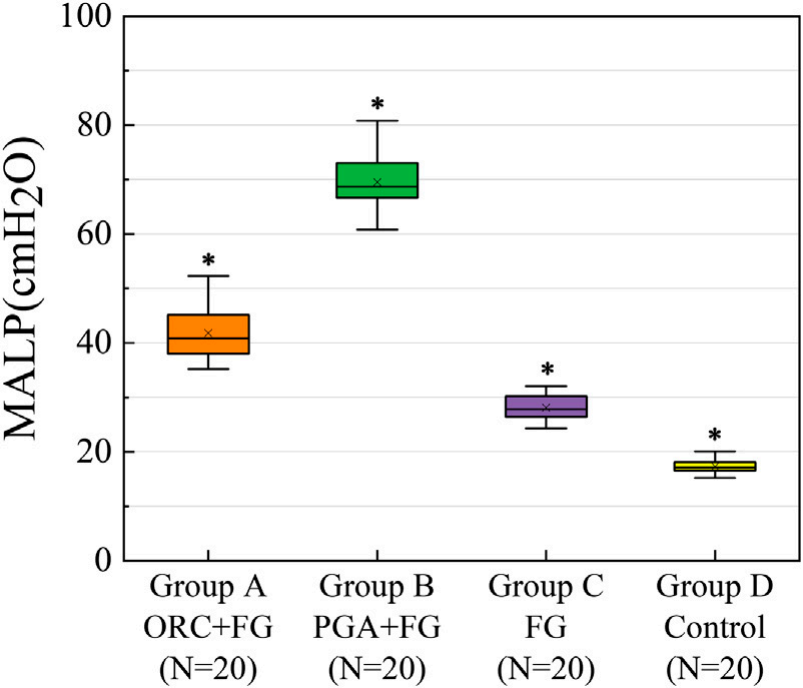
The MALP of the intersegmental plane in each experimental group. MALP was significantly higher in the group A, as compared with the group C and group D (both *p* < 0.001) with a significant difference between the latter two groups (*p* < 0.001). A significantly greater MALP was obtained for group B than the other groups (both *p* < 0.001). The upper and lower borders of the box represent, the upper and lower quartiles. The middle horizontal line represents the median. The cross represents the mean. The whiskers show the minimum and maximum values excluding the outliers. *p < 0.05, group A *versus* group B *versus* group C *versus* group D. MALP (minimum air leakage pressure), ORC (oxidized regenerated cellulose), PGA (polyglycolic acid), FG (fibrin glue).

The MALPs of the intersegmental plane in each experimental group were as follows: group A (n = 20): ORC + fibrin glue, 41.8 ± 4.5 cmH_2_O; group B (n = 20): PGA + fibrin glue, 69.5 ± 5.2 cmH_2_O; group C (n = 20): fibrin glue, 28.1 ± 2.3 cmH_2_O; group D (n = 20): control group, 17.3 ± 1.2 cmH_2_O (Figure 6). Furthermore, eight cases in group B showed no air leakage with maximum setting ventilator pressure (70.0 cm H_2_O). Therefore, additional pressure was manually applied in these cases.

One-way analysis of variance indicated a significant difference in the MALP of the four groups (F = 761.97, *p* < 0.001). Tukey’s test showed that the MALP was significantly higher in group A than in groups C and D (both *p* < 0.001), with a significant difference between groups C and D (*p* < 0.001), whereas a significantly higher MALP was obtained for group B compared to the other three groups (both *p* < 0.001).

In the intersegmental plane, the combined application of fibrin glue and the PGA patch had the strongest ability to prevent air leakage, followed by the combination of ORC and fibrin glue group. Fibrin glue could effectively improve the air leakage prevention ability of segmentectomy models, whereas the ORC or PGA sheet could significantly enhance the sealing capacity of fibrin glue.

### Histopathological findings of the porcine lung segmentectomy models

**FIGURE 7:**
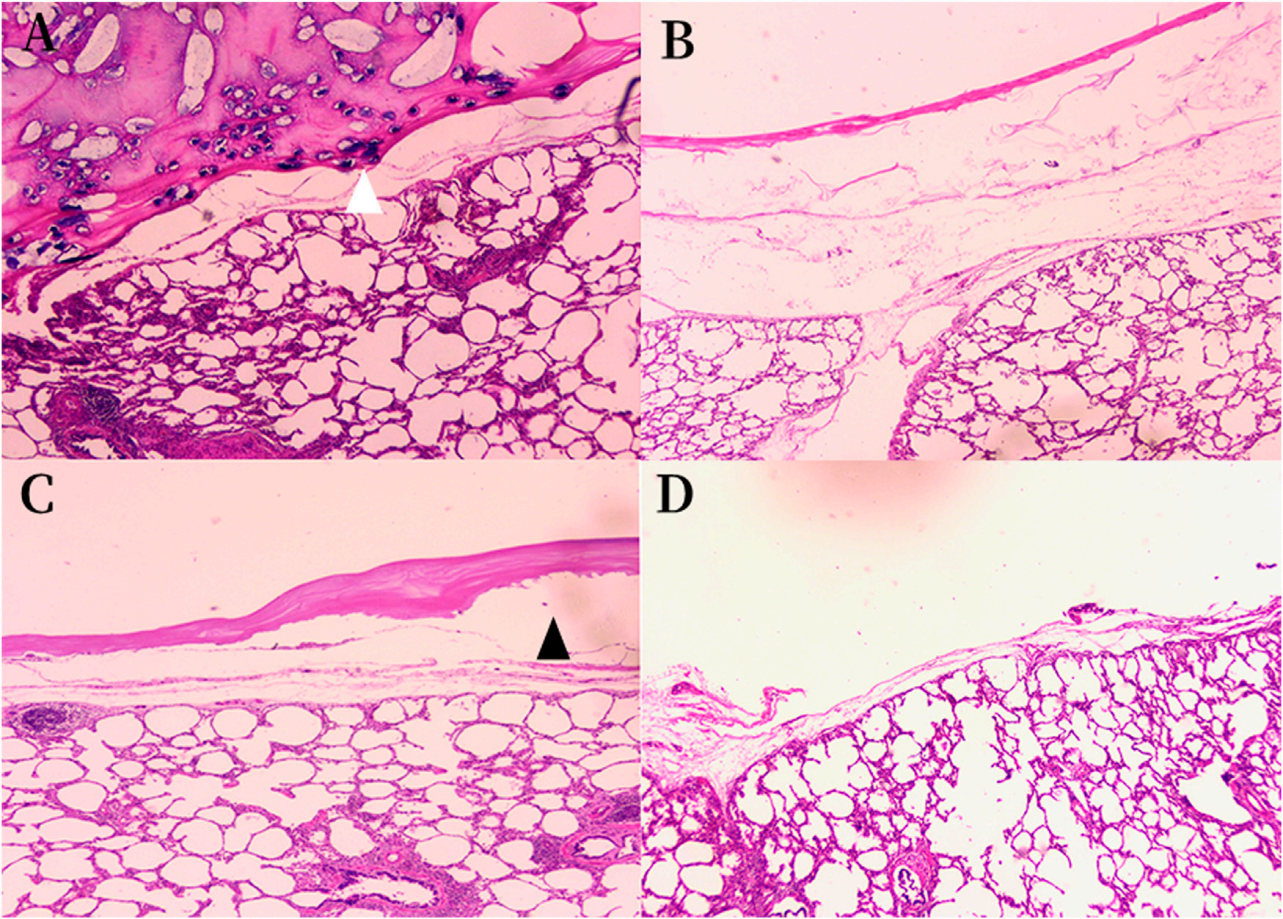
Histologic findings of the sealing materials on the intersegmental plane of the lung (original magnification ×40). **(A)** Group A ORC + fibrin glue, hematoxylin-eosin staining; **(B)** Group B PGA + fibrin glue, hematoxylin-eosin staining; **(C)** Group C fibrin glue, hematoxylin-eosin staining; **(D)** Group D control group, hematoxylin-eosin staining. **(A)** The sealing layer of ORC and fibrin glue adhered tightly to the intersegmental plane, some black gel blocked the pleural defect (white arrowhead). **(B)** The sealing layer of PGA and fibrin glue adhered firmly to the intersegmental plane. **(C)** A gap was observed between the sealing layer and the intersegmental plane (black arrowhead) in the group C. The sealing layer did not fit evenly with the surface of the lung tissue, and the thickness of it was not as uniform as that in groups A and B. **(D)** The normal intersegmental interface separated by electrocauterization. The pleural defect was severe due to the cauterization of the electric cautery. ORC (oxidized regenerated cellulose), PGA (polyglycolic acid).

In order to verify the adhesion strength microscopically, we performed histological examination on each group of experimental specimens. In the histological specimens of the porcine lung, hematoxylin-eosin staining revealed that the ORC and fibrin glue layer adhered tightly to the surface of the intersegmental plane in group A, while at the junction, cellulose was completely encapsulated by fibrin glue and some gel-like black substance could be seen near the pleural, which were formed by the reaction of the Fe^2+^ group in the hemoglobin with the carboxyl groups in the ORC to produce an acid-hematin complex. This black gel was tightly embedded in the sealing layer composed of ORC and fibrin glue and accumulated in large amounts in the pleural defect (Figure 7A).

The layer of PGA patch and fibrin glue in group B also adhered firmly to the interface of the lung segment (Figure 7B). However, there were some gaps between the fibrin glue and the surface of the lung segment in group C. The sealing layer did not fit evenly with the surface of the lung tissue, and the thickness of it was not as uniform as that in groups A and B. (Figure 7C). Conversely, in groups A and B specimens, no interspace was found between the lung tissue and the sealant layer. Figure 7D shows the normal intersegmental interface separated by electrocauterization. However, the pleural defect was severe due to the cauterization of the electric cautery.

## Discussion

According to the Japanese JCOG0802/WJOG4607L trial project results, segmentectomy provides a significantly better overall survival than lobectomy for patients with stage IA non-small cell lung cancer, suggesting that segmentectomy will become the standard treatment for early lung cancer in the future (Saji et al., 2022). However, air leakage was detected more in the segmentectomy arm than in the lobectomy arm (3.8% vs. 6.5%, *p* = 0.04), especially in patients undergoing complex segmentectomy or long-term smokers (Suzuki et al., 2019). Thus, how to reduce the incidence of air leakage after segmentectomy is an urgent clinical problem to be solved.

Clinically, sutures, staplers, and ligation are often used to close pulmonary air leaks; however, these methods may further reduce lung volume or cause further trauma to lung tissues (Saito et al., 2017) and have an indefinite effect in patients with fragile lung quality such as emphysema (Gika et al., 2007). Previous studies have shown that the combination of PGA and fibrin glue for covering the surgical area in pulmonary surgery can significantly decrease the occurrence of postoperative air leakage (Gika et al., 2007; Ueda et al., 2007; Kawai et al., 2012; Matoba et al., 2018; Tanaka et al., 2018); however, PGA sheet is expensive (Budak et al., 2020), and the method of covering the surgical wound with multi-layer PGA patch combined with fibrin glue proposed by Nomori et al. is difficult to popularize in clinical operations (Nomori et al., 2014). Furthermore, although PGA can be degraded completely, its degradation product, glycolide, can activate the classical complement pathway *in vivo*. Moreover, an inflammatory response to already weakened and stressed cells may result in significant cellular death and the failure of the implant of artificial materials (Ceonzo et al., 2006). Meanwhile, with the gradual replacement of lobectomy by segmentectomy, the number of patients undergoing secondary surgery due to ipsilateral recurrence or new-onset malignant tumors will increase, and the use of PGA sheets will be limited due to their tendency to cause thoracic adhesions (Nakamura et al., 2010; Saito et al., 2017). Therefore, identifying a substitute for PGA is critical.

Surgicel® is a widely used hemostatic material, and ORC is widely recognized as an effective agent of postoperative adhesion-prevention (Ten Broek et al., 2014). Several studies confirmed in animal model (beagle dogs) that ORC mesh is the preferred material for inducing increased thickening of the visceral pleura without inducing severe visceral-to-parietal pleural adhesions (Kurihara et al., 2016; Kanai et al., 2020; Yamanaka et al., 2019). Ebana et al. (2018) also confirmed this conclusion in clinical trials based on the detailed histopathological analyses of the resected lung specimens from five patients with recurrent pneumothorax after pleural covering with ORC mesh, and they found that 3 months after covering, the thickened pleura showed inflammatory cell infiltration, but those findings virtually disappeared after 1 year. Furthermore, we observed that postoperative air leakage in patients who underwent segmentectomy was reduced after the combined use of ORC and fibrin glue at the intersegmental plane in our center, implying that this method is a good choice for reducing postoperative air leakage. However, to our knowledge, few studies have been conducted on the use of ORC and fibrin glue for preventing pulmonary air leakage, and there is no recognized pleural covering technique to prevent air leakage after segmentectomy. Therefore, we designed a model of isolated porcine segmentectomy to test this conjecture.

To explore the effect of sealants in segmentectomy more realistically, we designed an *ex vivo* porcine segmentectomy model because of the similarity between the porcine lungs and human lungs (Judge et al., 2014). We observed that the MALP of the control group was (17.3 ± 1.2) cmH_2_O. After sealing the intersegmental plane with fibrin glue, the MALP reached (28.1 ± 2.3) cmH_2_O, which was significantly higher than that of group D, thus confirming the ability of the fibrin glue to prevent postoperative air leakage, in accordance with previous studies (Araki et al., 2007; Moser et al., 2008). However, this method is insufficient to meet the clinical need of segmentectomy. The ideal lung tissue sealant should be strong enough to withstand the expected lung expansion to 30–40 cmH_2_O pressure (Araki et al., 2007). Matoba et al., 2018 suggested that the sealant should provide 35 cmH_2_O strength to prevent air leakage under mechanical ventilation, and the pressure in the airway should be increased without causing air leakage when the patient has an irritative cough postoperatively. The MALP was 41.8 ± 4.5 cmH_2_O after combining ORC and fibrin glue in the intersegmental plane and 69.5 ± 5.2 cmH_2_O after combining PGA and fibrin glue, both of which were significantly higher than that of group C with fibrin glue alone. Thus, we can preliminarily conclude that the combination of ORC or PGA sheet with fibrin glue can effectively resist the increased airway pressure caused by cough, airway sputum blockage, or bronchospasm without causing postoperative air leakage. Therefore, this novel method of combining ORC with fibrin glue on intersegmental plane during operation can meet the demand for early airway pressure tolerance after segmentectomy.

In order to verify the adhesion strength microscopically, we performed a histological examination to verify the adhesion of different sealing materials. As shown in Figure 7, we observed that in group A, the sealing layer formed by ORC and fibrin glue adhered closely to the lung segment, while at the interface, we observed some black gel-like materials formed by the reaction of Fe^2+^ in the blood hemoglobin with the carboxyl groups in the cellulose to form acid-hematin (Wu et al., 2012). Therefore, we hypothesized that this black gel could help close the ruptured alveolar wall at the pleural defect. Furthermore, the sealing layer formed by the PGA patch and fibrin glue in group B also adhered tightly to the interface of the lung segment. In contrast, there were some gaps between the fibrin glue and the surface of the lung segment in group C, which did not exist in the sealing layer of groups A and B. The thickness of sealing layer in group C was not as uniform as that in groups A and B. Figure 7D shows the normal intersegmental interface separated by electrocauterization, the pleural defect was severe due to the cauterization of the electric cautery. Histological examination confirmed that both ORC and PGA could be combined with fibrin glue to enhance the sealing properties of fibrin glue.

Compared with the PGA sheet, the sealing effect of ORC is slightly weaker to combine with fibrin glue; however, the advantages of ORC are diverse, which include cheaper price and superior hemostatic properties. ORC also has the advantages of better extensibility and faster hydrolytic degradation in meeting certain tensile strengths. An elastic sealing layer material was used in group A with a uniform surface load that did not fall off with the expansion of target lung tissue. ORC is absorbed faster by the human body than PGA (7–14 days vs. 15 weeks) (Lewis et al., 2013; Kanai et al., 2020), and the degradation products are less toxic and are mostly oligosaccharides, which trigger minimal adverse reactions in the human body and have better biocompatibility (Dimitrijevich et al., 1990; Lewis et al., 2013). PGA is hydrolyzed to glycolic acid, then dimerized into glycolide, which is incorporated into the tricarboxylic acid cycle and eventually excreted by the kidneyd (Yang et al., 2005). Moreover, PGA and its degradation product, glycolide, can activate the classical complement pathway and cause inflammation *in vivo* (Ceonzo et al., 2006).

We used the classical pack method proposed by Gika et al. to apply the sealing materials in groups A and B (Gika et al., 2007). Previous studies have shown that the pack method has significantly higher seal-breaking pressure and better fibrin clot penetration into the tissue than other methods (Gika et al., 2007; Itano, 2008), possibly because the fibrinogen solution was 34.8 times more viscous and had 3.5 times higher osmolality than the thrombin solution (Itano, 2008). Thus, thrombin can easily penetrate the sheet, unlike fibrinogen.

In our study, the thrombin solution saturated the ORC sheet and then reacted with fibrinogen to form a stable fibrin clot in the tissue of the defect and adjacent intersegmental planes, which acted as a foothold that fixes the sealing material to the lung tissue. Furthermore, the fibrinogen solution can be absorbed by the sheet through capillary phenomena (Itano, 2008) to interact with the thrombin in the sheet fibers forming fibrin clots that fill the air spaces in the ORC sheet. In addition, the black gel mentioned above may help seal the pleural defect. Eventually, the sealing layer was integrated into a whole by adding the remaining fibrinogen and thrombin solutions on the felt, thereby contributing to excellent adhesion strength—histological findings of lung tissues in group A support this mechanism.

One limitation of this study is that these experiments were carried out using excised healthy pig lungs; thus, the MALP results cannot be applied directly to *in vivo* conditions or emphysematous lungs. In addition, a long-term study regarding the repair of pleural defects by ORC and PGA has not been performed.

In conclusion, we believe that ORC is a safe and low-cost material that can replace Neville® to combine with fibrin glue in the prevention and treatment of air leakage after segmentectomy. Our novel method has a strong sealing effect and good biocompatibility, prospects for use in clinical segmentectomy.

## Data Availability

The raw data supporting the conclusion of this article will be made available by the authors, without undue reservation.

## Abbreviations

Polyglycolic acid, (PGA); oxidized regenerated cellulose, (ORC); minimum air leakage pressure, (MALP); a and b segments of the right cranial lobe, (R1a, R1b); a and b segments of the left cranial lobe, (L1a, L1b); a and b segments of the left caudal lobe, (L2a, L2b).
